# Comparison of Metabolic Power and Energy Cost of Submaximal and Sprint Running Efforts Using Different Methods in Elite Youth Soccer Players: A Novel Energetic Approach

**DOI:** 10.3390/s24082577

**Published:** 2024-04-17

**Authors:** Gabriele Grassadonia, Pedro E. Alcaraz, Tomás T. Freitas

**Affiliations:** 1 UCAM Research Center for High Performance Sport, Universidad Católica de Murcia (UCAM), 30107 Murcia, Spain; gabriele.grassadonia@gmail.com (G.G.); palcaraz@ucam.edu (P.E.A.); 2 UPSS—International Department of Motor Arts, Popular University of Sport Sciences, 00122 Rome, Italy; 3 UPM—Department of Medical Sciences, Popular University of Milan, 20122 Milan, Italy; 4 MIU—Department of Sport Sciences, Miami International University, Miami, FL 33131, USA; 5 Faculty of Sport, Universidad Católica de Murcia (UCAM), 30107 Murcia, Spain; 6 Strength and Conditioning Society, 30008 Murcia, Spain; 7 NAR—Nucleus of High Performance in Sport, São Paulo 04753-060, Brazil

**Keywords:** football, maximum velocity, maximal running, GPS, EMG/force ratio

## Abstract

Sprinting is a decisive action in soccer that is considerably taxing from a neuromuscular and energetic perspective. This study compared different calculation methods for the metabolic power (MP) and energy cost (EC) of sprinting using global positioning system (GPS) metrics and electromyography (EMG), with the aim of identifying potential differences in performance markers. Sixteen elite U17 male soccer players (age: 16.4 ± 0.5 years; body mass: 64.6 ± 4.4 kg; and height: 177.4 ± 4.3 cm) participated in the study and completed four different submaximal constant running efforts followed by sprinting actions while using portable GPS-IMU units and surface EMG. GPS-derived MP was determined based on GPS velocity, and the EMG-MP and EC were calculated based on individual profiles plotting the MP of the GPS and all EMG signals acquired. The goodness of fit of the linear regressions was assessed by the coefficient of determination (R^2^), and a repeated measures ANOVA was used to detect changes. A linear trend was found in EMG activity during submaximal speed runs (R^2^ = 1), but when the sprint effort was considered, the trend became exponential (R^2^ = 0.89). The EMG/force ratio displayed two different trends: linear up to a 30 m sprint (R^2^ = 0.99) and polynomial up to a 50 m sprint (R^2^ = 0.96). Statistically significant differences between the GPS and EMG were observed for MP splits at 0–5 m, 5–10 m, 25–30 m, 30–35 m, and 35–40 m and for EC splits at 5–10 m, 25–30 m, 30–35 m, and 35–40 m (*p* ≤ 0.05). Therefore, the determination of the MP and EC based on GPS technology underestimated the neuromuscular and metabolic engagement during the sprinting efforts. Thus, the EMG-derived method seems to be more accurate for calculating the MP and EC in this type of action.

## 1. Introduction

The energy cost (EC) and kinematics of various forms of locomotion (e.g., running) have been analyzed in numerous investigations [1,2,3,4,5,6,7] with the aim of elucidating the main mechanisms of different movements. These studies have practical applications and allow for evaluating the metabolic energy expenditure or predicting the “ideal” performance [8,9,10,11,12,13,14] based on the relationship between mechanics and energetics [7,15,16,17,18,19], which is one of the most crucial and extensively researched domains of human movement [3,4,16,20,21,22,23,24,25,26,27].

For example, di Prampero et al. [22] estimated the EC of the first 30 m of a sprint running from a standing position to overcome the challenges of directly measuring effort during dynamic actions. In brief, the method relied on the equivalence between acceleration on flat ground and ascent at constant speed, with an equivalent slope defined by forward acceleration. Since the EC of constant speed on a varied range of slopes is well known [1,2,3,5,13,28], estimating the EC of the run is possible when the equivalence between forward acceleration and the slope is known. Therefore, di Prampero et al.’s [22] model has been suggested to redefine the concept of “high intensity”. Nevertheless, despite the new possibilities that arise from this approach [22] in terms of workload quantification and physical performance evaluation during training and competition [18,29,30,31,32,33,34], more evidence is still needed to determine the feasibility of EC estimation in applied scenarios.

Some researchers tried to evaluate neuromuscular and metabolic engagement during training and match events by using portable technology to understand muscle activation thresholds. A first attempt to characterize the profile of neuromuscular activation during a soccer match was proposed by Montini et al. [35], with the intention to integrate, in competition, more traditional laboratory-based approaches (e.g., electromyography [EMG]) to help better understand the physiological demands of competitive soccer. The authors analyzed different intensity zones to create a relative performance model and suggested that this approach could be used to improve the understanding of the physiological requirements of competitive soccer [35]. However, the EC and metabolic power (MP) calculated by EMG were not determined; thus, additional research is still necessary to consolidate measurements of economy and neuromuscular activation during performance activities that involve high-intensity running. This type of methodological approach is important for practitioners since, by using portable technologies, it is possible to collect data on more ecologically valid conditions than in laboratory settings.

The literature has explored the behavior of EMG during sprints and submaximal runs since Mero & Komi [36]; however, to the authors’ knowledge, it has not been utilized for the calculation of the MP and EC until Colli’s work (unpublished data retrieved from laltrametodologia.com). Thus, this remains a topic that needs further investigation to better understand the main mechanistic–energetic needs and, consequently, make meaningful methodological choices. Currently, there are numerous existing studies evaluating MP and energy expenditure, utilizing global positioning systems (GPS) and inertial measurement units (IMU) [24,32,33,37,38,39,40,41,42,43], but there is a complete absence of studies calculating these parameters from EMG technology. Analyzing submaximal and maximal sprint behavior with the aim of determining the MP and EC calculated by EMG and the EMG and force relationship could help clarify actual metabolic and neuromuscular engagement during linear running actions. The comparison of two distinct technologies (i.e., EMG and GPS-IMU) has the potential to provide precise estimates of relative effort for actions such as sprints, yielding hypothetical benefits.

Therefore, the aims of this study were to (1) analyze submaximal running efforts at various constant speeds to investigate possible differing mechanical–energetic demands when compared to sprinting; (2) examine the behavior of the EMG activity-to-force ratio (EMG/F) in linear sprints over 30 m and 50 m and their corresponding 5 m sections; and (3) determine the EC and MP of sprinting assessed by GPS-IMU and EMG by creating an ad hoc neuromuscular profile utilizing muscle activation patterns. The present study may have significant implications for the establishment and structuring of training objectives.

## 2. Materials and Methods

### 2.1. Study Design

**Figure 1:**
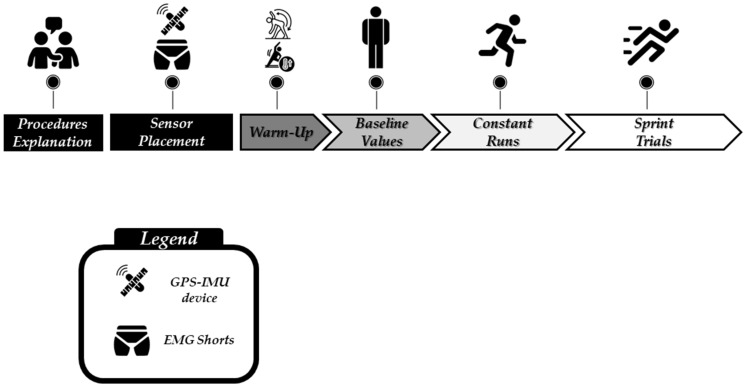
Overview of the study design.

A cross-sectional study design was used (Figure 1). Data were collected during the 2020/2021 competitive season, during the months of September through November, with players from the under-17 (U17) age category of a professional soccer club academy. To avoid a potential source of bias, de-identified data were analyzed by a researcher not directly involved in data collection. After a careful theoretical explanation accompanied by a practical demonstration, players completed four different submaximal constant running efforts followed by sprinting actions while using portable GPS-IMU units and surface EMG. All athlete measures were taken in a single testing session for each player during the pre-season period. The warm-up included mobility and running-based exercises for a duration of ~15 min. All warm-up exercises had been previously used by all the players, as they were applied in daily training.

### 2.2. Participants

A convenience sample of sixteen U17 football players (age: 16.4 ± 0.5 years; body mass: 64.6 ± 4.4 kg; height: 177.4 ± 4.3 cm; and BMI: 20.5 ± 1.3) of the “Elite Italian Championship” volunteered to participate in this study. A normal team practice and competition schedule, consisting of at least four training sessions and one match per week, was maintained during the investigation period. Only players who were free from recent injuries or medical conditions that could limit their maximum effort were included in the study. Detailed information regarding all testing and training procedures was provided to the subjects and their legal guardians before the latter signed a written informed consent. The Local Human Subjects Ethics Committee approved the study in compliance with the Declaration of Helsinki.

### 2.3. Procedures

#### 2.3.1. Constant Running and Sprint Testing

Four incremental constant (C_1,2,3,4_) running speeds (over 50 m, at theoretical required times of ~22.5, ~15, ~11.3, and ~9 s in “C_1_”, “C_2_”, “C_3_”, and “C_4_”, respectively) and a sprint effort (where only the split of the maximum speed phase was taken) were used for the construction of an individual profile (detailed below; coded with “S_5_”). Timing adherence was manually controlled using stopwatches during the constant runs in the trials. All tests were conducted on the training and match field, and each player was given the appropriate technical clothing to maintain their running characteristics (ecological field test). As mentioned, the players started by performing the constant runs with the objective of having an approximate constant difference between runs rather than a set datum (impossible for a field test that does not take place on an ergometer); thus, they were asked to maintain the same running characteristics during each trial. After the constant runs and a rest period (2 min), the players performed a total of three all-out sprints over 50 m. To establish the zone of maximum sprinting speed, a plateau with a delta of no more than 3 km·h^−1^ in the GPS data was selected to objectively determine when athletes reached their peak speed. A 5 min passive rest period was provided between trials to minimize fatigue effects on performance. Participants were encouraged to perform each sprint trial as fast as possible.

#### 2.3.2. Electromyography Recording and Analysis

**Figure 2:**
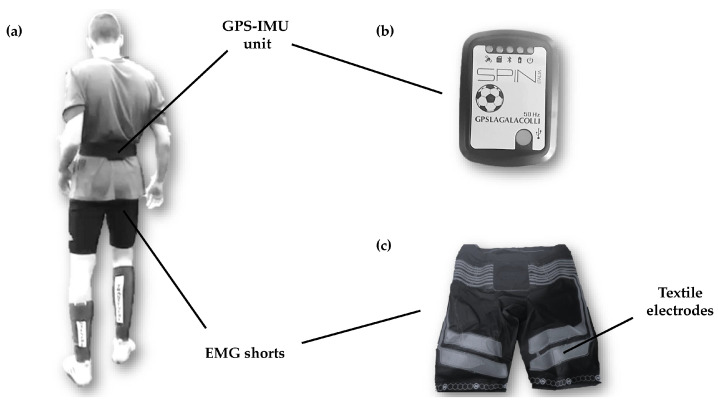
(**a**) Back view of the sensor placement; (**b**) GPS unit; (**c**) EMG shorts equipped with textile electrodes with 6 differential EMG biosignal channels.

During the trials, EMG shorts equipped with textile electrodes (Myonear Pro, Myontec, Kuopio, Finland) were used to collect muscle activation data (Figure 2). The conductive electrodes and the associated wires were integrated into the fabric. These electrodes covered three main muscle groups, bilaterally, with 6 differential EMG biosignal channels: quadriceps, hamstrings, and glutes. Two sizes of shorts were available (medium and large), and the best fit was chosen for each participant. The proper size of the shorts is essential to establish necessary contact between electrodes and skin and to minimize or avoid any movement artifacts during dynamic activities [44]. Additionally, a small amount of water was applied to the electrodes before the participant put on the shorts to ensure adequate signal conduction, as previously recommended [45]. EMG signals were transmitted to a laptop and analyzed and collected at 1000 Hz with the Myontec ‘’Muscle Monitor’’ software version 3.1.0.4 (Myontec Ltd., Kuopio, Finland). Textile electrodes embedded in shorts appeared to provide comparable lower limb muscle activation data to traditional surface EMG [44]. Each trial was firstly filtered with a second-order Butterworth band pass (a bandwidth of 40–200 Hz, derived through an exploration of the frequency domain with a signal voltage and −3 dB cutoff frequency) filter, before being rectified and averaged over 100 Hz. In accordance with Kyröläinen et al. [46], who criticized the use of voluntary maximum isometric contractions (MVICs) for evaluating neuromuscular activation during running, the EMG data were normalized using the peak EMG activity (EMGpeak) detected during the sprint, thus allowing for greater repeatability of the measurements. In addition, the EMG signals during the runs were segmented into subphases to enable a detailed analysis not only of the overall trend (total EMG recording [EMGTOT_ecf], comprehensive of ground contact, eccentric and concentric, and of the flight phases) but also of the characteristics of each phase (i.e., eccentric [EMGe], concentric [EMGc], and flight phase) utilizing the LagalaColli software (version 1.0.2.218, Spinitalia S.R.L., Rome, Italy). The EMG/F ratio was determined with an arbitrary unit, consisting of the ratio of the normalized EMG signal with peak values and expressing it as a percentage and the resulting force (in N·kg^−1^), which was calculated by integrating the accelerations from the three axes (x, y, and z) using IMU technology.

#### 2.3.3. Ad Hoc Profiling and Metabolic Power Calculation

Prior to the sprint analysis, an individual linear profile (including slope and intercept) was constructed for each athlete by plotting the MP of the GPS and muscle load (ML) from all the EMG signals acquired. The profile was individualized and made it possible to recalculate the MP from the EMG by a simple method that consisted of multiplying by the slope and then adding intercept (Equation (1)). Then, the EC was calculated by dividing the obtained value of MP by the speed achieved (Equation (2)).

(1) $${\mathrm{MP}}_{\mathrm{EMG}}=({\mathrm{ML}}_{\mathrm{EMG}}\cdot \mathrm{SLOPE})+I\mathrm{NTERCEPT}$$

(2) $${\mathrm{EC}}_{\mathrm{EMG}}=\frac{{\mathrm{MP}}_{\mathrm{EMG}}}{{\mathrm{SPEED}}_{\mathrm{IMU}}}$$

The EMG data were integrated with GPS-IMU signals to permit us to temporally and kinematically differentiate phases. Sprint analyses were conducted utilizing personalized spreadsheets. The GPS MP was based on the GPS velocity, and the integrated GPS-IMU velocity was utilized to determine the EMG MP. The GPS data were recorded at 50 Hz and the IMU at 100 Hz in accordance with the manufacturer’s instructions.

### 2.4. Statistical Analysis

Statistical analyses were conducted using the Statistical Package for Social Sciences (SPSS) software, version 25.0 (Chicago, IL, USA), Microsoft Excel 2019 (Redmond, WA, USA), and the free Statistical Software Jamovi 2.3.28. Data are presented as means and standard deviations. The goodness of fit of the linear regressions was assessed by the coefficient of determination (R^2^) and the confidence interval (CI, set at 95%). The Shapiro–Wilk test was used to verify if the values were normally distributed, and the Wilcoxon signed rank nonparametric test was used for data not normally distributed. A repeated measures ANOVA was used to detect changes, with a two-sample F-test for variances. The effect size (ES, Cohen’s d) of the intervention was calculated using Cohen’s guidelines [47,48]. The threshold values for the ES were small (≥0.2), medium (≥0.5), and large (≥0.8). For all procedures, a level of *p* ≤ 0.05 was selected to indicate statistical significance.

## 3. Results

**Figure 3:**
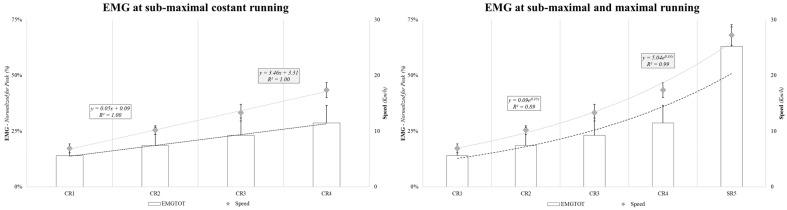
EMG at four different submaximal running speeds (**left** panel) and with sprint efforts (**right** panel).

During the experimental period, no injuries were sustained by any of the players, and the compliance with the assessments and degree to which the participants adhered to the study protocol and accepted the interventions and assessments were maximal, as there were no dropouts. Regarding the study results, these include the EMG signal and speed mean values, detected with the EMG signal in a bipodal static (2.4 ± 0.6% relative to the EMG_peak_) and obtained during the four incremental constant running and sprint efforts over 50 m. The first constant running (C_1_) exercise was completed at 6.9 ± 0.8 km·h^−1^, with an EMG_e_ of 16.2 ± 8.3%, an EMG_c_ of 14.0 ± 3.8%, and an EMG_TOT_ecf_ of 13.9 ± 5.3%. In the second constant running (C_2_) exercise, the speed was 10.2 ± 0.8 km·h^−1^ with an EMG_e_ of 21.6 ± 11.8%, an EMG_c_ of 17.7 ± 6.4%, and an EMG_TOT_ecf_ of 18.5 ± 8.3%. C_3_ was completed at 13.3 ± 1.5 km·h^−1^, with EMG_e_, EMG_c_, and EMG_TOT_ecf_ values of 27.4 ± 11.8%, 23.0 ± 5.9%, and 23.1 ± 7.8%, respectively. Finally, the speed reached in C_4_ was 17.4 ± 1.3 km·h^−1^, with an EMG_e_ of 33.1 ± 8.7%, an EMG_c_ of 30.4 ± 12.6%, and EMG_TOT_ecf_ of 28.6 ± 8.0%. Regarding the sprint effort (S_5_), the speed achieved was 27.3 ± 1.8 km·h^−1^, the EMG_e_ was 62.3 ± 8.6%, the EMG_c_ was 57.4 ± 8.6%, and the EMG_TOT_ecf_ 63.1 ± 8.7%. Figure 3 displays the corresponding total EMG patterns in relation to running speed.

**Figure 4:**
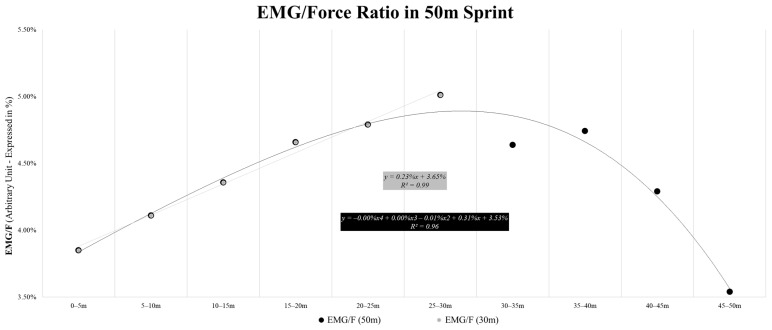
Linear (gray) and curvilinear (polynomial, in black) fits of the EMG/F ratio over 50 m sprints.

During sprinting, the EMG/F ratio data were processed for each 5 m interval (Figure 4) and presented a double behavior interpolated with two types of fit. The EMG/F ratio was linear up to 30 m (R^2^ = 0.99) and polynomial (fourth degree) up to the completion of 50 m (R^2^ = 0.96).

**Figure 5:**
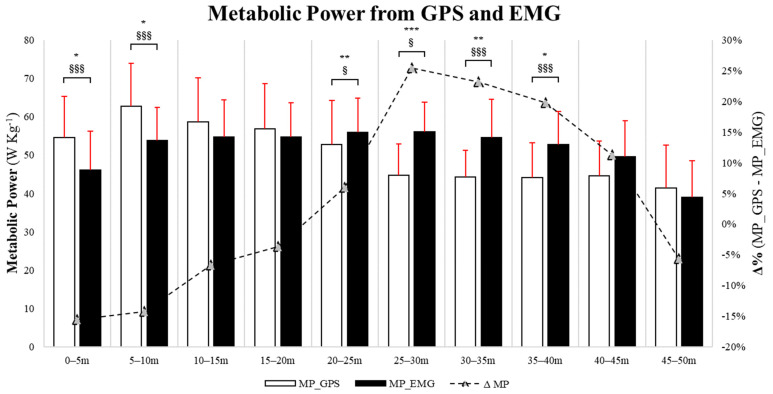
Metabolic power calculated via GPS and EMG methods during the linear 50 m sprint. * *p*-value ≤ 0.05, ** *p* ≤ 0.01, *** *p* ≤ 0.001; § ES ≥ 0.20, §§ ES ≥ 0.50, §§§ ES ≥ 0.80.

Comparisons of the MP for the different split distances obtained with the GPS and EMG are presented in Figure 5. The data were not normally distributed at 20–25 m and 35–40 m distances. In the 0–5 m and 5–10 m splits, the MP calculated with the GPS was significantly higher than with EMG (0–5 m: *p* = 0.03, F = 1.13, ES = 0.81; 5–10 m: *p* = 0.02, F = 1.67, ES = 0.89). Conversely, in the 10–15 m and 15–20 m ranges, no significant differences were observed between both methods (10–15 m: *p* = 0.31, F = 1.42, ES = 0.37; 15–20 m: *p* = 0.58, F = 1.76, ES = 0.20; 20–25 m: *p* = 0.39, F = 1.66, ES = 0.10). In the 20–25 m, 25–30 m, 30–35 m, and 35–40 m splits, the MP was significantly lower when determined via the GPS rather than EMG (20–25 m: *p* = 0.01, F = 1.66, ES = 0.31; 25–30 m: *p* ≤ 0.001, F = 1.17, ES = 1.42; 30–35 m: *p* = 0.002, F = 0.48; ES = 1.19; and 35–40 m: *p* = 0.02, F = 1.15, ES = 0.98). Lastly, no differences between the MP determined via the GPS and EMG were found in the 40–45 m (*p* = 0.14; F = 0.94, ES = 0.54) and 45–50 m splits (*p* = 0.53; F = 1.38, ES = 0.22). Table S1 (in Supplementary Files) illustrates the values obtained after adjustments, applying the nonparametric statistical test.

**Figure 6:**
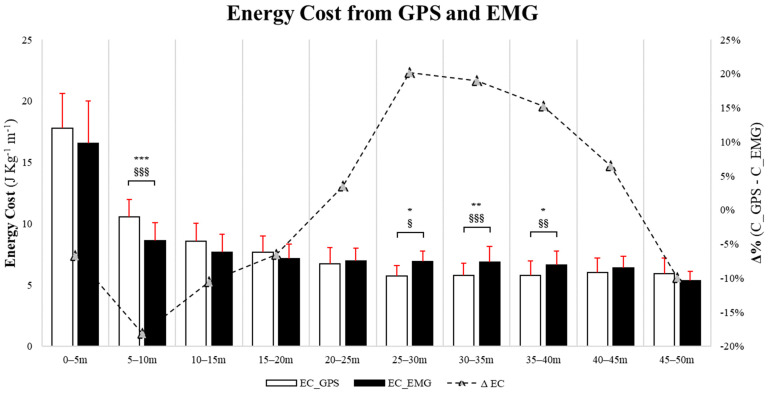
Energy cost calculated via GPS and EMG methods during the linear 50 m sprint. * *p*-value ≤ 0.05, ** *p* ≤ 0.01, *** *p* ≤ 0.001; § ES ≥ 0.20, §§ ES ≥ 0.50, §§§ ES ≥ 0.80.

The EC estimated through the GPS and EMG is displayed in Figure 6. The data were not normally distributed at the distances 15–20 m, 20–25 m, 25–30 m, 30–35 m, and 35–40 m. No differences were found between both approaches in the 0–5 m split (*p* = 0.30, F = 0.68, ES = 0.38), which contrasts with the 5–10 m split, in which the EC determined via the GPS was significantly greater (*p* = 0.001, F = 1.03, ES = 1.33). In the 10–15 m (*p* = 0.09, F = 1.03, ES = 0.63), 15–20 m (*p* = 0.09, F = 1.32, ES = 0.40), and 20–25 m (*p* = 0.54, F = 1.66, ES = 0.20) ranges, no differences in EC were identified. Regarding the 25–30 m (*p* = 0.02; F = 0.96, ES = 1.35), 30–35 m (*p* = 0.003, F = 0.61, ES = 0.95), and 35–40 m (*p* = 0.05, F = 1.14, ES = 0.76) ranges, the EC estimated through EMG was significantly higher. Finally, in the 40–45 m (*p* = 0.30, F = 1.57, ES = 0.37) and 45–50 m splits (*p* = 0.12, F = 2.78, ES = 0.56), no differences were observed in the EC estimated with the GPS and EMG. Table S2 (in Supplementary Files) illustrates the values obtained after adjustments, applying the nonparametric statistical test.

## 4. Discussion

The present study aimed to investigate the EMG activity and the EMG/F ratio during different submaximal and maximal runs and to study the differences between GPS-IMU and EMG technologies in the calculation of the MP and EC during constant running and sprinting efforts. The main findings indicate a linear increase in EMG values with running speed during the submaximal runs, which becomes exponential when considering the inclusion of sprinting. Moreover, the current results demonstrate the existence of a linear increase in the EMG/F ratio in sprints up to a breaking point (i.e., observed at 30 m) when an alteration in the overall trend is observed (i.e., considering the whole 50 m). In addition, differences were found at certain splits between the MP and EC calculated from the GPS-IMU and EMG, which indicates that these technologies cannot be used interchangeably to determine these metrics. Taking this into account, the present findings suggest that EMG seems to be a more precise technology for accurately estimating the MP and EC, showing a higher EC for sprinting, especially at greater speeds.

Notably, in the constant-speed runs, the EMG activity increased linearly with increasing speed. However, when also considering the sprint actions, the best-fitting trend becomes exponential (Figure 3). This might have an important implication for the study of the metabolic engagement of running efforts, as it supports the idea that sprint situations may cause an important increase in the energy expenditure of soccer players [36,46,49,50]. However, it should be considered that the present study did not specifically consider the EC of acceleration, high-speed running, and deceleration efforts, although from previous studies [3,12,22,33], we could already hypothesize significant differences between these types of actions. From a coaching perspective, the results herein could be used to determine the effective energetic and neuromuscular engagement needed for different types of actions and better understand performance models to develop the best training methodologies.

Another important parameter to be considered when investigating the interaction between external and internal loads during actions such as linear sprinting is the EMG/F ratio [51,52,53,54,55]. The current data highlight that this ratio appears to linearly increase until a ‘breakpoint’, where a decrease occurs (i.e., at 30 m, as evidenced by the data interpolation in Figure 4), which may have significant practical applications. In brief, it indicates that the expression of neuromuscular parameters likely varies across different distances and sports contexts, providing practitioners with an ideal range of distances that could be used in sprint training. For example, for youth soccer players, from an energetic and neuromuscular perspective, it may not be optimal to perform linear sprints greater than 30 m due to the observed decline in EMG/F. In our analyzed sample, there appears to be difficulty in maintaining the neuromuscular engagement characteristics indicated by the EMG/F marker over longer distances. This may be due to poor sprinting habits for longer distances, particularly under static start conditions. Future studies should verify the behavior of this parameter in other populations of athletes (e.g., sprinters), assuming that the drop in the EMG/F ratio should be postponed as much as possible for those who must perform linear sprints.

Finally, based on the construction of an ad hoc profile that allowed for the calculation of the MP and EC from EMG, it appears that the GPS-IMU approach may systematically underestimate the actual cost of sprinting in a statistically significant manner, especially for sprint actions between 25 and 40 m, when compared to EMG. This may be explained, at least in part, by the fact that at higher speeds, acceleration rates are considerably lower [49,56] but more costly; hence, the GPS-IMU may not be the most appropriate approach to quantify energy expenditure. Of note, EMG technology seems to display different MP and EC engagement with a much more “curvilinear pattern” (i.e., a fourth-degree polynomial relationship) during sprints, thus emphasizing a different, realistically more accurate engagement in some splits. These considerations could be useful for coaches and physical trainers to understand actual energy engagement and neuromuscular parameters in soccer, knowing more about its limitations and potential [56,57]. However, further research is required to determine the practical applications of this area of study in different populations with different purposes. In accordance with Van Hooren et al. [6], the calculation of the markers would be important for optimizing energetic and mechanical efficiency, possibly minimizing injury occurrence resulting from internal (i.e., physiology) and external (i.e., environment) sources. All these findings seem to be important in characterizing sprint action. The MP and EC analyses using EMG in comparison to the GPS may provide more precise results for evaluating neuromuscular and metabolic activity. This approach can be advantageous for optimizing mechanical–energetic requirements and warrants further investigation, including cognitive engagement during exercises involving a ball.

This study has several limitations that should be considered when interpreting the results. Firstly, the cross-sectional design used prevents us from drawing any causal inferences regarding the examined variables. Secondly, only isolated and “decontextualized” linear sprints without a ball were assessed when it is known that, in soccer, most physical capacities are expressed along with technical–tactical elements with the ball [32,38]. Thus, the data here should not be directly extrapolated to sprinting during soccer matchplay. Thirdly, other important running-based actions, such as accelerations and decelerations, were not assessed and compared in detail. Additionally, the EC and MP were estimated through a GPS-IMU and EMG, and the use of a portable gas analyzer could have enhanced the study’s accuracy, providing practical assistance and a better understanding when comparing data. This was demonstrated by Savoia et al. [33] when comparing the GPS algorithm based upon di Prampero’s theoretical model in elite soccer players with a measure obtained with a portable gas analyzer. Nevertheless, the methodological approach here is more practical and easier to apply in real-world contexts, which is an important point worth highlighting. Further research is necessary to determine whether and how the current findings may be affected by training adaptations.

The outcomes of this study may be useful for strength and conditioning coaches to plan their sessions more effectively. Our data examined the EC of running at different speeds and identified the EMG trends indicative of actual neuromuscular demands. The analysis of an internal-to-external load ratio, such as the EMG/F ratio, may be useful in determining appropriate distances for training. In addition, the differences between the MP and EC calculated by the GPS-IMU and EMG suggested an important underestimation of the actual demands of high-speed actions by the former (which must be considered when developing training exercises). However, it is important to note that the current data were collected from a sample of U17 soccer players from a Mediterranean context and that the generalization of the results to other populations should be made cautiously. Further research should be conducted to investigate these aspects and potential disparities in game scenarios.

## 5. Conclusions

In summary, this study presents a new perspective for characterizing running activities in soccer, utilizing parameters such as the EMG/F ratio and using the MP and EC calculated from EMG, and just a GPS-IMU. Defining and characterizing the specifics of physical engagement are strategic factors for designing a novel approach [58,59,60] to study neuromuscular and metabolic activity to continue development [3,33,49,56]. Although additional research is necessary, these indicators appear suitable for accurately studying workload, improving performance, examining the dose–response relationship of exercise, and identifying the onset and modification of fatigue during competitions. In the future, a GPS-IMU and EMG should be validated against direct measures of energy expenditure, both external and internal, to determine their relationship with direct measures of fitness and performance.

## Data Availability

Data are contained within the article.

## Supplementary Materials

The following supporting information can be downloaded at https://www.mdpi.com/article/10.3390/s24082577/s1: Table S1: MP; Table S2: EC.
