# Supplementary material for: Comparison of Metabolic Power and Energy Cost of Submaximal and Sprint Running Efforts Using Different Methods in Elite Youth Soccer Players: A Novel Energetic Approach
Source: Sensors (Basel). 2024 Apr 17;24(8):2577. doi: 10.3390/s24082577 (PMC11054392; doi:10.3390/s24082577)
Supplement: Supplementary file 1 [file sensors-24-02577-s001.zip › sensors-2946480-supplementary.pdf]

sensors

MDPI

Supplementary files for

Comparison of Metabolic Power and Energy Cost of Submaximal and Sprint Running Efforts Using Different Methods in Elite Youth Soccer Players: A Novel Energetic Approach

Gabriele Grassadonia, Pedro E. Alcaraz and Tomás T. Freitas

MP

Splits	*Mean MP_GPS*	*SD MP_GPS*	*Mean MP_EMG*	*SD MP_EMG*	*CV (%) MP_GPS*	*CV (%) MP_EMG*	*p-value*	*ES*	*F*	*CI (95%, lower)*	*CI (95%, upper)*
0-5m	54.58	10.80	46.07	10.15	19.79%	22.04%	0.029*	0.81§§§	1.13	2.54	14.465
5-10m	62.77	11.23	53.81	8.70	17.89%	16.16%	0.017*	0.89§§§	1.67	2.56	15.381
10-15m	58.61	11.57	54.70	9.70	19.74%	17.74%	0.308	0.37§	1.42	-4.65	12.478
15-20m	56.88	11.83	54.78	8.91	20.80%	16.27%	0.575	0.20§	1.76	-3.89	8.086
20-25m	52.73	11.53	55.89	8.94	21.86%	16.00%	0.01**	0.31§	1.66	-11.72	4.195
25-30m	44.67	8.31	56.07	7.70	18.60%	13.73%	0.000***	1.42§§§	1.17	-15.88	-6.901
30-35m	44.25	6.97	54.51	10.04	15.75%	18.42%	0.002**	1.19§§§	0.48	-14.54	-5.982
35-40m	44.08	9.17	52.79	8.56	20.81%	16.22%	0.015*	0.98§§§	1.15	-14.59	-2.23
40-45m	44.53	9.17	49.58	9.46	20.59%	19.08%	0.136	0.54§§	0.94	-10.6	0.519
45-50m	41.35	11.20	39.02	9.54	27.08%	24.46%	0.532	0.22§	1.38	-2.67	7.318

Table S1. Metabolic Power outcomes and statistical analysis results for the different splits. CV = Coefficient of Variation; EC = Energy cost; ES = Effect size; SD = Standard deviation; * p-value$\leqslant$0.05, ** $p \leqslant 0.01$, *** $p \leqslant 0.001$; §§ ES $\geqslant$ 0.20, §§ ES $\geqslant$ 0.50, §§§§ ES $\geqslant$ 0.80.

EC

Splits	*Mean EC_GPS*	*SD EC_GPS*	*Mean EC_EMG*	*SD EC_EMG*	*CV (%) EC_GPS*	*CV (%) EC_EMG*	*p-value*	*ES*	*F*	*CI (95%, lower)*	*CI (95%, upper)*
0-5m	17.76	2.85	16.57	3.46	16.04%	20.87%	0.296	0.38§	0.68	-1.23	3.62
5-10m	10.54	1.45	8.64	1.42	13.71%	16.49%	0.001***	1.33§§§	1.03	0.84	2.97
10-15m	8.59	1.45	7.69	1.43	16.89%	18.59%	0.087	0.63§§	1.03	-0.30	2.10
15-20m	7.67	1.33	7.17	1.16	17.32%	16.11%	0.085	0.40§	1.32	-0.50	1.38
20-25m	6.72	1.33	6.95	1.03	19.84%	14.86%	0.542	0.20§	1.66	-1.32	0.69
25-30m	5.73	0.85	6.89	0.86	14.76%	12.52%	0.02*	1.35§§§	0.96	-1.76	-0.55
30-35m	5.78	1.00	6.87	1.29	17.38%	18.71%	0.003**	0.95§§§	0.61	-1.58	-0.61
35-40m	5.76	1.19	6.64	1.12	20.69%	16.80%	0.048*	0.76§§	1.14	-1.57	0.08
40-45m	6.02	1.16	6.41	0.92	19.20%	14.39%	0.298	0.37§	1.57	-1.04	0.26
45-50m	5.94	1.28	5.35	0.77	21.56%	14.36%	0.124	0.56§§	2.78	-0.15	1.33

Table S2. Energy Cost outcomes and statistical analysis results for the different splits. * p-value$\leqslant$0.05, ** $p \leqslant 0.01$, *** $p \leqslant 0.001$; § ES $\geqslant$ 0.20, §§ ES $\geqslant$ 0.50, §§§ ES $\geqslant$ 0.80.
